# Seasonal dynamics of arboreal spider diversity in a temperate forest

**DOI:** 10.1002/ece3.221

**Published:** 2012-04

**Authors:** Yu-Lung Hsieh, Karl Eduard Linsenmair

**Affiliations:** Department of Animal Ecology and Tropical Biology, Universität WürzburgD-97074 Würzburg, Germany

**Keywords:** Araneae, canopy fogging, European beech, recolonization, species richness estimation, true diversity

## Abstract

Measuring and estimating biodiversity patterns is a fundamental task of the scientist working to support conservation and inform management decisions. Most biodiversity studies in temperate regions were often carried out over a very short period of time (e.g., a single season) and it is often—at least tacitly—assumed that these short-term findings are representative of long-term general patterns. However, should the studied biodiversity pattern in fact contain significant temporal dynamics, perhaps leading to contradictory conclusions. Here, we studied the seasonal diversity dynamics of arboreal spider communities dwelling in 216 European beeches (*Fagus sylvatica* L.) to assess the spider community composition in the following seasons: two cold seasons (I: November 2005–January 2006; II: February–April) and two warm seasons (III: May–July; IV: August–October). We show that the usually measured diversity of the warm season community (IV: 58 estimated species) alone did not deliver a reliable image of the overall diversity present in these trees, and therefore, we recommend it should not be used for sampling protocols aimed at providing a full picture of a forest's biodiversity in the temperate zones. In particular, when the additional samplings of other seasons (I, II, III) were included, the estimated species richness nearly doubled (108). Community I possessed the lowest diversity and evenness due to the harsh winter conditions: this community was comprised of one dominant species together with several species low in abundance. Similarity was lowest (38.6%) between seasonal communities I and III, indicating a significant species turnover due to recolonization, so that community III had the highest diversity. Finally, using nonparametric estimators, we found that further sampling in late winter (February–April) is most needed to complete our inventory. Our study clearly demonstrates that seasonal dynamics of communities should be taken into account when studying biodiversity patterns of spiders, and probably forest arthropods in general.

## Introduction

Increased destruction and disturbance of natural habitats has strengthened the need to understand biodiversity patterns and their spatial and temporal variation for the purpose of supporting conservation and management decisions (Hsieh et al. 2003). Currently, most biodiversity studies, especially rapid assessments, rely on intensive sampling over only short-time periods. These studies however, while certainly informative, often—at least implicitly—assume that such short-term results can be generalized and taken as representative of long-term patterns (Hughes et al. 2002; Shahabuddin et al. 2005). However, those studies paying attention to seasonal variation have revealed that it may play a very important role in species turnover, and thus, its contribution to overall biodiversity may be underappreciated (Thomas and Thomas 1994; Leps et al. 1998; Summerville and Crist 2003). This critically challenges our understanding of biodiversity patterns and requires analyses along temporal scales (Tylianakis et al. 2005; Hsieh and Linsenmair 2011a): if species’ distributions are aggregated in time due to strong seasonality, the time frame of sampling could lead to serious under- or overestimation of diversity and might result in contradictory or misleading inferences and conclusions.

In temperate forests there are large seasonal variations in temperature. For this reason it is essential to carry out biodiversity sampling over an extended period comprising all seasons—in given a year at least—to clarify the basics pattern of temporally variable diversity (Floren and Schmidl 2008). Tree crowns in the temperate zone offer an excellent opportunity to investigate temporal variations, because the fauna is cleared annually by winter's onset and we can measure the appearance and disappearance of spiders across changing seasons. This cycle of colonization and recolonization could be described by the theory of island biogeography (MacArthur and Wilson 1963, 1967), which might describe in a basic way the seasonal community dynamics of arboreal arthropods in canopies of temperate and higher latitude forests. The theory predicts that the number of species on a true island or habitat island, such as tree crowns in this case (Müller and Goßner 2007), results from the dynamic relationship between local immigrations and extinctions. Thus, by studying arboreal arthropods in these temperate forest canopies, we may examine temporal dynamics of species richness and their seasonal contribution to overall biodiversity.

Among arthropods, spiders are a valuable surrogate for assessing predatory arthropod diversity and for studying general spatial and temporal biodiversity patterns (Marc et al. 1999; Platnick 1999; Cardoso et al. 2004). This is because spiders are among the most species-rich animal orders (Coddington and Levi 1991; Nyffeler 2000), including about 42,751 described species with an estimated total of about 170,000 species (Coddington and Levi 1991; Platnick 2012), and they contribute significantly to abundance and diversity of terrestrial arthropods (Platnick 1999). Their study has implications not only for biodiversity and conservation issues but also for the timing and availability of ecosystem services facilitated by arboreal spiders: namely, catching great quantities of insects as prey in temperate regions (Nyffeler and Benz 1987; Nyffeler 2000). Furthermore, shifts in spiders’ guild composition can also be used to monitor the habitat change (Brown 2003), to assess microclimate complexity (Downes et al. 1998; Gunnarsson et al. 2004), and perhaps lead to an effective niche separation (Cerda et al. 1998).

The systematic study reported here examined the species richness, diversity, evenness, and similarity (or species turnover) of arboreal spider communities at different temporal (seasonal) scales over a 1-year period. We independently aggregated abundance data from the Würzburg University Forest, Germany, at different temporal focal scales. We asked the following questions: (1) Does species richness, diversity, and guild composition of communities in different seasons show strong temporal variation? (2) Which families and guilds characterize the process of recolonization in the warm seasons? (3) Based on nonparametric estimators, how many additional individuals would be needed to complete comprehensive inventories of spider diversity at our forest site?

## Materials and Methods

### Study area

**Figure 1:**
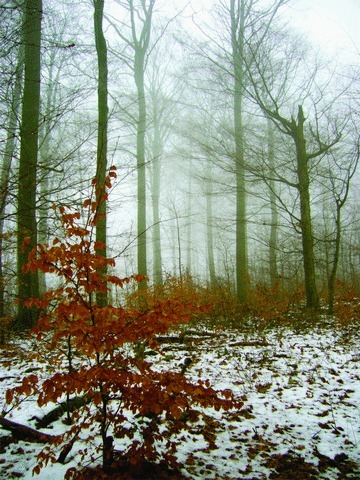
The Würzburg University Forest (Bavaria, Germany) is dominated by European beech (*Fagus sylvatica* L.). Tree crowns in the temperate zone offer an excellent opportunity to investigate temporal variation, because the fauna is cleared annually by winter's onset and the appearance and disappearance of arboreal spiders can be measured across changing seasons.

We conducted our study in the Würzburg University Forest, northern Bavaria, Germany (50°01¢N, 10°30¢E), where the mean yearly temperature and precipitation is 7.5°C and 675 mm. This temperate forest is dominated by European beech (*Fagus sylvatica* L., Fig. 1.) and we studied three of managed beech patches. Six replicates (individual beeches) were sampled per patch in this study on a monthly basis from November 2005 to October 2006 (i.e., 18 new beeches per month), and the spider communities of these 216 beeches (unrepeated samples) served as basis for studying seasonal variation in tree canopies.

### Canopy sampling

Beech canopies were fogged with pyrethrum at daybreak using a fogging machine (Swingfog™ SN-50). The fogging lasted approximately 10 min followed by a 2-h dropping time. Dropping arboreal arthropods were subsequently collected on plastic sheets previously positioned on the ground (588 m^2^ for 18 trees per month). Spiders were sorted for identification to species level by using species-specific attributes of the palpal organ and/or epigynum using the keys provided by Heimer and Nentwig (1991) and Roberts (1996); nomenclature follows Platnick (2012).

### Data analyses

Collecting biodiversity data is labor intensive and time consuming (Longino and Colwell 1997; Lawton et al. 1998). A substantial fraction of a community is often represented by many rare species, often singletons, which remain undetected by most biodiversity surveys (Chao et al. 2009). Since rare species usually contain information about incomplete sampling (Preston 1962a, b), we used nonparametric estimators to control undersampling bias (Colwell and Coddington 1994; Beck and Schwanghart 2010). These are based on frequency count and information on the rare species in the collection to estimate “true diversity” (Jost 2006, 2007): (1) Estimates of total species richness (the true diversity of order zero) was estimated by the Chao1 estimator (Chao 1984; Shen et al. 2003; Chao et al. 2006), which uses the numbers of singletons and doubletons to estimate the missing species’ numbers because information on missing species is mainly derived from rare species. (2) Exponential Shannon index (the true diversity of order 1) was measured by the Jackknife method (Zahl 1977). (3) Inverse Simpson index (the true diversity of order 2) was calculated using an approximate minimum variance unbiased estimator (MVUE, Magurran 1988). The abundance-based data were analyzed with SPADE (Species Prediction And Diversity Estimation, Chao and Shen 2003). We also give the 95% confidence intervals (CIs) to define the sampling variation, constructed using 200 bootstrap replications (Chao 1987; Chao et al. 2006).

SPADE was also used to estimate the similarity (or species turnover) of spider communities between seasons. The abundance-based Morisita index can investigate the degree of association in the distribution and recolonization of spiders between seasons. In order to predict the additional amount of sampling needed to reach 100% of the asymptotically increasing species richness, we generated subsamples from the fogging data using the Chao1 estimator (Chao et al. 2009). We also predicted the probability that the next sampled individual represents a previously undetected species in a further survey for future sampling planning. In addition, the distribution of a particular guild among different seasons can be used to assess seasonal environmental change and to point to which guild might drive recolonizations. Chi-square goodness-of-fit tests were used to examine whether or not certain guilds were more pronounced in a particular season.

## Results

### Structure and seasonal pattern of arboreal spider communities

In total 10,675 individuals were collected from 216 fogged beeches. Owing to the large proportion of juveniles, only 4305 spiders were able to be identified to 78 species belonging to 14 families (Table 1, Appendix). Anyphaenidae made up the largest portion of individual spiders collected (34.3%), followed by Tetragnathidae (15.6%), Araneidae (15.4%), Linyphiidae (14.1%), Theridiidae (12.3%), and Thomisidae (3.9%). Linyphiidae had the greatest number of species (33), followed by Araneidae and Theridiidae (10), Tetragnathidae (6), Clubionidae, Salticidae, and Thomisidae (3). The most abundant species was *Anyphaena accentuata*; a total of 1478 individuals were found (34.3%). The second most abundant species was *Metellina mengei* (*n*= 544, 12.6%), followed by *Neriene peltata* (*n*= 362, 8.4%), *Paidiscura pallens* and *Mangora acalypha* (*n*= 251, 5.8%), *Cyclosa conica* (*n*= 243, 5.6%).

**Table 1:** Number of families, species richness, unique species, rare species (singletons and doubletons), and abundances of arboreal spiders in beech trees (*Fagus sylvatica* L.) in four seasonal communities in Germany. Two cold seasons (I: November 2005–January 2006; II: February–April) and two warm seasons (III: May–July; IV: August–October).

Season	I	II	III	IV	Overall
Family	6	9	12	11	14
Species	12	27	52	41	78
Unique species	0	7	23	14	44
Singleton	5	11	16	13	22
Doubleton	1	1	8	5	8
% Rare species	50	44.4	46.2	43.9	38.5
Abundance	438	291	796	2780	4305

In order to analyze temporal dynamics, we categorized the four seasonal patterns of spider communities: two cold seasons (community I: November–January; II: February–April) and two warm seasons (III: May–July; IV: August–October). Thirty-six sets of beeches were used to analyze seasonal preferences of spiders, with each set consisting of six spider communities (i.e., six beech trees) collected from the same patch in the same month: community IV possessed higher abundance of arboreal spiders (308.89 ± 103.06 [mean ± SE, *n*= 9 sets]), whereas communities I (48.67 ± 18.55), II (32.33 ± 7.52), and III (88.44 ± 17.62) had fewer individuals (one-way analysis of variance [ANOVA]: *F*_3,32_= 5.818, *P* < 0.01, Tukey's pairwise comparisons: IV > I = II = III). Only six species were constantly active in all four seasons (*A. accentuata, M. mengei, P. pallens, Diaea dorsata Hyptiotes paradoxus*, and *Erigone atra*), whereas 44 species (56.4%) were only collected in one seasonal community and showed clear seasonal preferences. Table 1 shows that up to 60 species were possibly seasonally rare, that is, comprising one or two individuals only were found during one season (45 singletons and 15 doubletons). However, when pooling all four seasonal communities, the number of rare species is divided by 2, thus reaching only 30 species (22 singletons and eight doubletons).

### Estimates of arboreal spider diversity and seasonal similarity

Table 2 shows that at least 108 species were expected by the individual-based Chao1 estimator at species saturation, whereas only 58 species were expected in community IV (samples of the months from August to October). In the cold seasons, 25 species were expected in community I, but 88 species in community II. The calculation based on Chao1 also predicted the additional sample size needed to detect 100% of the estimated species richness: it varied from 4225 individuals (community III) to 19,257 individuals (IV) in different seasons. Overall, an additional 35,578 individuals would need to be collected and identified to reach the full set of local species. Moreover, it was predicted with higher probability (4%) that in a next survey the next sampled individual in community II would represent a previously undetected species.

**Table 2:** Estimation of species richness, diversity, and evenness (95% confidence interval) in four seasonal communities on forest beech trees in Germany.

	I	II	III	IV	Overall

**Estimated species richness (true diversity of order 0)**					
Chao1	25	88	68	58	108
% Inventory completion	48	30	76.5	70.7	72.2
*P*	0.01	0.04	0.02	<0.01	0.01
*N*	5347	10,770	4225	19,257	35,578
**Exponential Shannon entropy (true diversity of order 1)**					
Jackknife	1.56	11.14	13.91	10.29	12.61
	(1.37, 1.76)	(9.46, 12.83)	(12.43, 15.39)	(9.84, 10.74)	(12.04, 13.17)
**Inverse Simpson index (true diversity of order 2)**					
Approximate MVUE	1.17	6.88	7.28	6.74	6.4
	(−0.43, 2.77)	(6.38, 7.37)	(6.84, 7.72)	(6.2, 7.28)	(5.85, 6.94)

*P*, the probability that the next sampled individual represents a previously unseen species. *N*, the number of additional individuals needs to reach estimated richness.

The exponential Shannon entropy estimate showed that seasonal community III had the greatest diversity (13.91 [12.43, 15.39]), whereas community I possessed the lowest diversity (1.56 [1.37, 1.76]). The inverse Simpson index showed that the spider assemblage in community I (November–January) was most influenced by dominant species (1.17 [−0.43, 2.77]). The abundance-based Morisita indices showed that the global similarity between the four seasons was 52.3% with a 95% CI of (48.4, 56.2); furthermore, similarity was significantly lower (38.6% [33.1, 44.0]) between communities I and III (Table 3).

**Table 3:** Pairwise similarity (%) with a 95% confidence interval among four seasonal communities studied on 216 beech forest trees in Germany.

Season	I	II	III	IV

I	100			
II	52.9 (44.5, 61.3)	100		
III	38.6 (33.1, 44.0)	55.5 (48.4, 62.5)	100	
IV	55.4 (52.6, 58.2)	72.0 (65.8, 78.2)	63.7 (57.8, 69.5)	100

### Seasonal preference of arboreal spider guild

**Figure 2:**
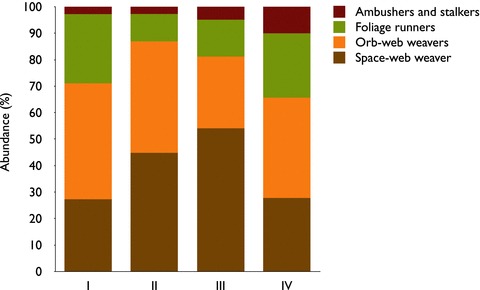
Arboreal spider guild composition of four seasonal communities on 216 European beech trees (*Fagus sylvatica*) in Germany based on pooled monthly samples taken over a 1-year period.

A total of 9673 spiders were able to be identified to 15 families, and four spider guilds were analyzed: space-web weavers (Dictynidae, Linyphiidae, and Theridiidae, 33.8%); orb-web weavers (Araneidae, Tetragnathidae, and Uloboridae, 37.3%); foliage runners (Anyphaenidae and Clubionidae, 21.6%); ambushers and stalkers (Philodromidae, Salticidae, and Thomisidae, 7.4%) (modified from Uetz et al. 1999). Amaurobiidae, Gnaphosidae, Lycosidae, and Sparassidae were excluded for this analysis owing to their low abundances. Chi-square tests demonstrated two seasonal patterns in guild composition (Table 4): community II and III consisted of a large proportion of space-web weavers and a small proportion of foliage runners (Fig. 2). In contrast, communities I and IV consisted of relatively more foliage runners and fewer space-web weavers.

**Table 4:** Arboreal spider guild composition between each seasonal community examined by values (lower-left triangle) and significance levels (upper-right triangle) of chi-square tests of homogeneity.

Season	I	II	III	IV

I		<0.05	<0.01	0.33
II	11.11		0.26	<0.01
III	17.02	5.24		<0.01
IV	4.58	13.98	14.83	

## Discussion

Our study is among the first to study arboreal spider diversity at the canopy strata on a year-round temporal scale. The 216 European beeches were fogged over 12 consecutive months. Although some passive sampling techniques (e.g., branch traps) are much easier to run over long periods, fogging in winter may perform better for studies of the biodiversity dynamics of arboreal spiders because many winter-active arthropods stay hidden in marcescent leaves. As suspected, the results revealed that the community structure and diversity of arboreal spiders changed with seasons. Since no prior study has attempted to determine whether spiders can survive the harsh conditions of the canopy in winter, the study contains novel data from the canopies of deciduous trees in the temperate zone. Using individual tree canopies as experimental units, we can analyze the effects of recolonization and disappearance on local biodiversity assemblages.

### Seasonal community structure and diversity of arboreal spiders

Some insect communities in temperate forests also contain species that are aggregated due to host plant phenology (Summerville and Crist 2003; Veech et al. 2003; Summerville and Crist 2005). However, the distribution of spiders is mostly governed by microclimate, vegetation architecture, prey availability, as well as oviposition site availability (Halaj et al. 2000). Low diversity and abundance in community I pointed to the obvious fact that the harshest winter conditions for arboreal spiders were from November to January, such that only two species (*A. accentuate* [55 individuals] and *H. paradoxus* [1]) were found on 18 beech trees in January. The low value of the inverse Simpson index suggests that community I (November–January) was dominated by the former species (92%, 405 of 438 individuals), and this relatively high abundance of *A. accentuata* in community I is surprising. Indeed, this species is known to require specific habitats to hide from predation pressure, given that tree crowns usually start to lose their leaves in November. Most deciduous woody plants in the temperate zones shed their leaves in late autumn and go through the entire winter leafless. However, many European beeches exhibit marcescence (Fig. 1); that is, they retain their dry leaves through winter and shed them only the following spring (Otto and Nilsson 1981). This phenomenon might have a significant effect on microclimate during harsh winters and seems to provide a very important refuge for winter-active spiders. The distribution of spiders exhibits associations with the marcescence phenomenon; thus, *A. accentuata* became a consistent and ubiquitous colonizer and its high abundance in these winter trees can prevent its local extinction (i.e., rescue effect; Brown and Kodric–Brown 1977; Pulliam 1988). This phenological trait of European beeches might attract winter-active spiders that become sedentary, with the effect of controlling herbivorous insects, especially in the winter and following spring under milder climate allowing spider activity. Moreover, winter-active spiders might also be a relevant food resource for insectivorous species (e.g., *Sitta europaea* and *Dendrocopus medius*) particularly in temperate winter forests. Our finding indicates that beech marcescence is as important as evergreen trees (e.g., *Pinus sylvatica*) for winter-active arthropods and points to its conservation value. We therefore suggest that marcescent beeches not be overlogged or disturbed during winter to avoid biodiversity loss of spiders.

From February onward, however, the spider communities were no longer dominated by *A. accentuata* (as suggested by the higher inverse Simpson index value). The families that characterized the process of recolonization and contributed to forming the community II were mainly Amaurobiidae, Araneidae, Salticidae, and some members of Linyphiidae, which did not occur in community I. These families were important in structuring the following warm seasonal communities because being so abundant there was little “extinction” risk (in the island sense) in the warm seasons. Conversely, the small populations of Dictynidae and Philodromidae found in community III stand the greatest chance of becoming locally “extinct” in the canopy “island.” But owing to the transient families, community III was well mixed and thus had the highest diversity. After recolonization communities II and III had significantly different guild compositions compared to community I, and the least similarity between communities III and I further confirmed they were heterogeneous due to higher species turnover.

Although there were no significant differences in the diversity and dominance between communities II and IV, higher species richness was estimated for community II (February–April). This is due to the fact that many space-web weavers started to immigrate into sparse canopies in April (early spring) before the tree crowns were fully covered by fresh leaves. This increased proportion of space-web weavers suggests they are important in spider recolonization in spring months: most of their members (e.g., Linyphiidae and Theridiidae) are quite small and disperse easily in warm seasons (e.g., by ballooning, Bonte et al. 2008); thus, they exerted a quick and strong effect on the restructuring of the communities. And while communities II and III both contained a large proportion of space-web weavers, many of these species disappeared locally from the canopies in community IV (August–October) and many likely went “extinct”. A further reason for the latter's disappearance might be that some species left the tree, below to the ground in order to search for mating opportunities or suitable oviposition sites (Draney 1997). In parallel, some species which had returned to tree canopies since August (e.g., *Drapetisca socialis* and *M. segmentata*) were found as late as December, but not from January to July. After 6 months, the similar species and guild compositions between communities IV and I shows that community IV had re-established itself and become identical to the original community I (last winter composition). This return confirms that communities in the temperate zone were not stable, but in a state of flux. This is likely a characteristic of the beech forest canopy communities and may be essential for maintaining ecosystem function (as prey and predator), particularly during winter.

### True diversity estimates and sufficient sampling

Using the Chao1 nonparametric estimator, we made point estimates and direct comparisons between seasons even if the seasonal communities contained different numbers of spiders. The estimator predicted that there are in total 108 arboreal spiders in beech forests within the Würzburg University Forest. The Chao1 estimator showed different estimates of species richness (25–88 species) among seasons, indicating that bias can be generated when only sampling in one single season in a given year, because phenology causes many species to be rare at most time periods (Summerville and Crist 2003, 2005). Since Chao1 only uses the numbers of rare species to estimate the number of missing species (Chao et al. 2006), our study, which takes seasonal variability into account, and thus brings the number of rare species to half its seasonal value (seasonal: 60 rare species; overall: only 30), better avoids the overestimation and represents the true diversity of arboreal spiders in the European beech forest.

Sufficient sampling is important in order to study biodiversity and make proper conservation decisions. The estimation of species richness according to the Chao1 estimator predicted that over 70% of the available taxa had been sampled in communities III and IV; however, 52% and 70% of the available taxa had not been sampled in communities I and II, respectively. This shows that substantial sampling is still needed to survey the remaining species during the cold season months (November–April). In short, it is probably impossible to detect all expected species without taking the cold season into explicit account. Further, there is a higher probability of finding an undetected species from February to April (late cold season). Thus, the cold months, which harbor a significant amount of undetected rare species and offer the relevant information for estimating true diversity, require additional focus. We anticipate that such investigations will prompt us to revise species richness and biodiversity studies in the temperate forest ecosystems.

Months from August to October are most suitable for many spiders compared to other months, as shown by higher abundance of spiders in this season (community IV). However, the diversity estimate obtained from data of those months alone is an underestimate: it failed to yield a reliable picture of the beech forest canopies’ diversity. If the sampling had been restricted this season only, we would have considerably underestimated the species richness (58 species) to just over half (54%) of the actual estimated species richness over a year's sampling. Based on our results, we can claim that the composition and diversity of arboreal spider communities in temperate forests is strongly influenced by seasonal variables. We should not only focus on the partitioning of spatial components of community structure (Hsieh and Linsenmair 2011b), but also take into consideration temporal factors when studying biodiversity. In this way, current estimates of the number of arthropod species may increase, or even double as shown here, thus enabling us to gain a more accurate image of canopy diversity in forest trees.

## Appendix

**Table A1:** Monthly abundance of the 78 spider species observed in European beeches in Germany over a 1-year period of sampling on 216 trees.

Taxon	2005		2006									
	N	D	J	F	M	A	M	J	J	A	S	O
**Amaurobiidae**	.	.	.	.	.	.	.	.	.	.	.	.
*Amaurobius fenestralis*	.	.	.	.	1	.	.	.	.	.	.	.
*Coelotes terrestris*	.	.	.	.	.	.	.	.	.	1	.	.
**Anyphaenidae**	.	.	.	.	.	.	.	.	.	.	.	.
*Anyphaena accentuata*	191	159	55	11	2	70	47	25	89	211	307	311
**Araneidae**	.	.	.	.	.	.	.	.	.	.	.	.
*Araneus diadematus*	.	.	.	.	.	.	.	.	.	1	1	1
*Araneus sturmi*	.	.	.	.	.	.	.	9	.	54	.	84
*Araneus triguttatus*	.	.	.	.	.	.	3	.	1	.	.	.
*Araniella cucurbitina*	.	.	.	.	.	.	.	.	1	.	.	.
*Araniella opisthographa*	.	.	.	.	.	.	.	2	.	.	.	.
*Cyclosa conica*	.	.	.	30	15	.	13	6	.	110	.	69
*Gibbaranea bituberculata*	.	.	.	.	.	.	.	1	.	4	.	4
*Gibbaranea gibbosa*	.	.	.	2	1	.	.	.	.	.	.	.
*Mangora acalypha*	.	.	.	.	.	.	.	1	.	207	.	42
*Zilla diodia*	.	.	.	.	.	.	1	1	.	.	.	.
**Clubionidae**												
*Clubiona caerulescens*	.	.	.	.	.	.	1	.	.	.	.	.
*Clubiona comta*	.	.	.	.	.	.	.	8	.	2	.	2
*Clubiona terrestris*	.	.	.	.	.	.	.	.	.	2	.	.
**Dictynidae**												
*Lathys humilis*	.	.	.	.	.	.	1	.	.	.	.	.
*Nigma flavescens*	.	.	.	.	.	.	16	7	1	.	.	.
**Linyphiidae**												
*Araeoncus humilis*	.	.	.	.	.	1	2	.	.	.	.	.
*Bathyphantes gracilis*	1	.	.	.	.	1	.	.	.	1	.	.
*Cnephalocotes obscurus*	.	.	.	.	.	1	.	.	.	.	.	.
*Dicymbium nigrum*	.	.	.	.	.	.	.	.	.	.	.	1
*Diplocephalus picinus*	.	.	.	.	.	.	.	1	.	.	.	.
*Drapetisca socialis*	4	2	.	.	.	.	.	.	.	12	9	3
*Entelecara acuminata*	.	.	.	.	.	.	1	11	.	1	.	.
*Entelecara erythropus*	.	.	.	.	.	1	.	1	.	.	.	.
*Erigone atra*	1	3	.	.	.	19	1	.	5	.	1	1
*Erigone dentipalpis*	.	1	.	.	.	5	.	.	.	.	.	.
*Erigonella hiemalis*	.	.	.	.	.	3	.	1	.	.	.	.
*Labulla thoracica*	.	.	.	.	.	.	.	.	.	.	1	.
*Lepthyphantes flavipes*	.	.	.	.	.	.	.	.	.	1	.	.
*Lepthyphantes minutus*	.	.	.	.	.	.	.	.	.	1	.	.
*Linyphia hortensis*	.	.	.	.	.	.	2	.	.	.	.	.
*Linyphia triangularis*	.	.	.	.	.	.	.	.	.	9	.	.
*Maso sundevalli*	1	.	.	.	.	.	.	1	.	.	.	.
*Meioneta rurestris*	2	.	.	.	.	9	.	3	3	.	.	.
*Mermessus trilobatus*	.	.	.	.	.	12	1	2	.	1	.	2
*Moebelia penicillata*	.	.	.	.	.	.	.	.	.	2	2	.
*Neriene emphana*	.	.	.	.	.	.	.	.	2	1	.	.
*Neriene peltata*	.	.	.	9	16	.	48	27	2	180	.	80
*Obscuriphantes obscurus*	.	.	.	.	.	.	.	1	.	.	.	.
*Oedothorax apicatus*	.	.	.	.	.	.	.	.	4	.	.	.
*Porrhomma microphthalmum*	.	.	.	.	.	9	1	4	1	.	.	1
*Porrhomma oblitum*	.	.	.	.	.	.	.	2	.	.	.	.
*Porrhomma pygmaeum*	.	.	.	.	.	.	.	.	1	.	.	.
*Saaristoa firma*	.	.	.	.	.	1	.	.	.	.	.	.
*Sintula corniger*	.	.	.	.	1	.	.	.	.	.	.	.
*Tenuiphantes tenebricola*	.	.	.	.	.	.	.	3	.	.	.	.
*Tenuiphantes tenuis*	.	.	.	.	.	.	.	.	.	2	.	.
*Thyreosthenius parasiticus*	.	.	.	.	3	.	.	.	.	.	.	.
*Troxochrus nasutus*	.	.	.	.	.	47	8	1	.	.	.	.
**Lycosidae**												
*Pardosa saltans*	.	.	.	.	.	.	2	.	1	.	.	.
**Philodromidae**												
*Philodromus albidus*	.	.	.	.	.	.	.	3	.	.	.	.
*Philodromus collinus*	.	.	.	.	.	.	.	.	1	.	.	.
**Salticidae**												
*Ballus chalybeius*	.	.	.	1	.	.	.	5	2	19	2	33
*Evarcha falcata*	.	.	.	.	.	.	.	.	.	1	.	.
*Heliophanus cupreus*	.	.	.	.	.	.	.	.	.	1	.	.
**Sparassidae**												
*Micrommata virescens*	.	.	.	.	.	.	.	.	.	.	.	1
**Tetragnathidae**												
*Metellina mengei*	4	.	.	.	.	2	3	55	9	242	21	208
*Metellina segmentata*	.	1	.	.	.	.	.	.	.	1	3	2
*Pachygnatha degeeri*	.	.	.	.	.	1	.	.	.	.	.	2
*Tetragnatha montana*	.	.	.	2	2	.	.	.	.	.	.	.
*Tetragnatha obtusa*	.	.	.	.	.	.	.	.	4	.	.	.
*Tetragnatha pinicola*	.	.	.	.	.	.	.	.	.	50	.	60
**Theridiidae**												
*Achaearanea lunata*	.	.	.	.	.	.	.	1	.	.	.	.
*Achaearanea simulans*	.	.	.	.	.	.	.	.	4	2	.	.
*Anelosimus vittatus*	.	.	.	.	.	.	.	2	.	1	.	.
*Enoplognatha ovata*	.	.	.	.	1	.	.	33	17	1	1	1
*Keijia tincta*	.	.	.	.	.	.	2	13	8	97	1	27
*Paidiscura pallens*	2	3	.	1	2	2	94	94	27	17	6	3
*Robertus arundineti*	.	.	.	.	.	.	1	.	.	.	.	.
*Theridion mystaceum*	.	.	.	.	.	.	1	1	.	17	.	27
*Theridion pinastri*	.	.	.	.	.	.	.	.	1	12	1	.
*Theridion varians*	.	.	.	.	.	.	.	6	1	.	.	.
**Thomisidae**												
*Diaea dorsata*	1	.	.	2	4	.	3	4	1	81	1	68
*Synaema globosum*	.	.	.	.	.	.	.	1	.	.	.	.
*Xysticus lanio*	.	.	.	.	.	.	1	.	2	.	.	.
**Uloboridae**												
*Hyptiotes paradoxus*	1	5	1	.	.	1	1	.	18	11	26	8
